# Presenting a codon-optimized palette of fluorescent proteins for use in *Candida albicans*

**DOI:** 10.1038/s41598-020-63308-w

**Published:** 2020-04-09

**Authors:** Wouter Van Genechten, Liesbeth Demuyser, Peter Dedecker, Patrick Van Dijck

**Affiliations:** 1 Laboratory of Molecular Cell Biology, Institute of Botany and Microbiology, KU Leuven, 3001 Leuven Belgium; 2 VIB-KU Leuven Center for Microbiology, KU Leuven, 3001 Leuven Belgium; 3 Department of Chemistry, KU Leuven, 3001 Leuven Belgium

**Keywords:** Protein folding, Proteomics, Super-resolution microscopy, Fungal biology

## Abstract

Fluorescent proteins with varying colors are indispensable tools for the life sciences research community. These fluorophores are often developed for use in mammalian systems, with incremental enhancements or new versions published frequently. However, the successful application of these labels in other organisms in the tree of life, such as the fungus *Candida albicans*, can be difficult to achieve due to the difficulty in engineering constructs for good expression in these organisms. In this contribution, we present a palette of *Candida*-optimized fluorescent proteins ranging from cyan to red and assess their application potential. We also compare a range of reported expression optimization techniques, and find that none of these strategies is generally applicable, and that even very closely related proteins require the application of different strategies to achieve good expression. In addition to reporting new fluorescent protein variants for applications in *Candida albicans*, our work highlights the ongoing challenges in optimizing protein expression in heterologous systems.

## Introduction

*Candida albicans* is an opportunistic human fungal pathogen. This pathogen is able to cause relatively harmless superficial infections of the skin and mucosa or severe invasive bloodstream infections with a mortality rate of 46 to 75%^1^. Because of the limited amount of antifungal drug classes available and the diverse set of resistance mechanisms against existing antifungals, research on this pathogenic fungus is necessary in order to discover novel targets for antifungal therapy^2^. Molecular research on yeast species such as *C. albicans* relies on biochemical techniques and tools that were often first developed for use in the model organism *Saccharomyces cerevisiae* or mammalian cell systems. For example; bimolecular fluorescence complementation (BiFC) was first applied in COS-1 cells and has only recently been introduced in *C. albicans* by our group^3,4^. Even simple plasmids from the closely related yeast *S. cerevisiae* have to be adapted because of the instability of episomal plasmids in *C. albicans* and the CUG codon, which is translated as serine instead of leucine in 97 out of 100 cases^5,6^. Heterologous expression of genes in *C. albicans* requires additional manipulations, such as specific codon adaptations due to the previously described CUG codon, but also due to differing overall codon usage of *C. albicans* compared to other fungi^7^.

Even in *Escherichia coli and S. cerevisiae*, where significant enhancements in synthetic biology were already made, acquiring a functional and highly expressed heterologous protein often requires the construction and screening of large libraries of codon variants^8,9^. Furthermore, the selection of a specific strain for overexpressing a protein can play a role in protein solubility and expression levels, since codon bias-adjusted strains do not always improve protein folding^10^. There is a clear need for a better understanding of codon usage and its application in heterologous expression.

Light microscopy is one of the indispensable tools in cell biology to investigate processes on a molecular scale. The use of fluorescent proteins in combination with microscopy or flow cytometry allows for the visualization or quantification of biological molecules and processes inside the cell. The first and only complete *Candida-*specific optimized fluorescent protein is γmGreen Fluorescent Protein (γmGFP). This FP was first codon-optimized in 1997, where each individual codon was replaced. This optimization was guided by a codon usage table that tabulated the frequencies of each codon in three highly expressed genes; *TEF3, ENO1* and *HSP70*^11^. Later adaptations were made to the amino acid sequence of the original *Candida* optimized GFP. The three resulting GFP variants were denoted α, β and γ, of which the γmGFP variant has improved folding properties, signal intensity and photostability^12^. However, these probes have essentially the same emission spectrum, which means that e.g. multi-color imaging is currently difficult. Because these tools are essential for research on *C. albicans* and accelerate the discovery of novel antifungal targets, we sought to further obtain optimized fluorescent proteins covering more of the visual spectrum.

A close variant of GFP is Yeast enhanced monomeric Venus (YemVenus), which is based on the original *S. cerevisiae* codon-optimized YeVenus combined with the A206K mutation to render it monomeric^13^. Thus, the YemVenus that was used in previous research on *C. albicans* was in fact codon optimized for *S. cerevisiae*^3,14^. This is also the case for the Cyan Fluorescent Protein (CFP), which gives a low overall fluorescent signal when used in *C. albicans* (WVG and LD, personal communications, 2018). We therefore selected a rationally improved version of CFP called mTurquoise2, which has a fluorescence quantum yield of 93% and improved photostability, for further codon optimization^15^. Whilst these previous FPs are all related to the classical GFP isolated from *Aequorea victoria*, red fluorescent proteins (RFP) were isolated from *Anthozoa* species. However, most of these RFPs tend to dimerize and have inferior brightness. Recently, mScarlet was engineered together with two variants mScarlet-I and mScarlet-H. We selected mScarlet-I for this work, because of its improved maturation speed^16^. Together with the advent of super-resolution microscopy methods, such as PALM^17^, RESOLFT^18^ and pcSOFI^19^, came the quest for novel and improved photoswitchable fluorescent proteins (PSFP). These proteins are able to reversibly switch between a dark ‘off’ state and a bright ‘on’ state. One of these proteins is the green fast-forming (ff)Dronpa, which has proven suitable for sub-diffraction imaging^20^.

Using these five different fluorescent proteins, we assessed different codon optimization techniques to obtain a library of fluorescent protein coding variants resulting in improved *Candida albicans* specific versions for each colour. Analysing several properties of the DNA sequences *in silico* shows a trend between folding of the mRNA sequence and the expression of each variant.

## Results

### *In silico* design of codon variants

We explored a number of different *in silico* optimization tools. The first optimization method is provided by Integrated DNA Technologies, Inc. (IDT, Illinois, USA). This tool uses a random number generator to select codons based on the frequency in the organism’s codon table, thereby eliminating codons with frequencies below 10%. It also removes complexities in the sequence, such has high GC content and repeats. The OPTIMIZER method similarly relies on a guided random selection of codons, using a Monte Carlo algorithm to select codons from a codon usage table^21^. The codon usage table for *C. albicans* was already available on the OPTIMIZER server and applied to obtain the OPTIMIZER entire genome sequences (OPT Entire). Another interesting feature of this tool is the possibility of using a codon usage set by tabulating highly expressed genes. This method was applied in the previously published optimization and application of pHluorin2 in *C. albicans*, where they selected *RPL29, RPL32, RPL39, ACT1* and *ENO1* to form the reference codon usage table^22^. We further refer to this optimization as OPT High. For the fourth optimization method, we applied the ATGme tool, which provides a bulk optimization feature that simultaneously exchanges all rare codons of a certain amino acid for the most frequently used codon^23^, thereby establishing a codon sequence with the highest Codon Adaptation Index (CAI). Lastly, a closely related protein to γmGFP such as mTurquoise2 and YemVenus, can benefit from having a codon sequence that is similar to γmGFP. We therefore manually adapted the codon sequence of γmGFP to insert mutations that are specific to mTurquoise2 and YemVenus, thereby obtaining γmGFP based fluorescent proteins.

### Brightness assessment: fluorescence activated cell sorting

**Figure 1:**
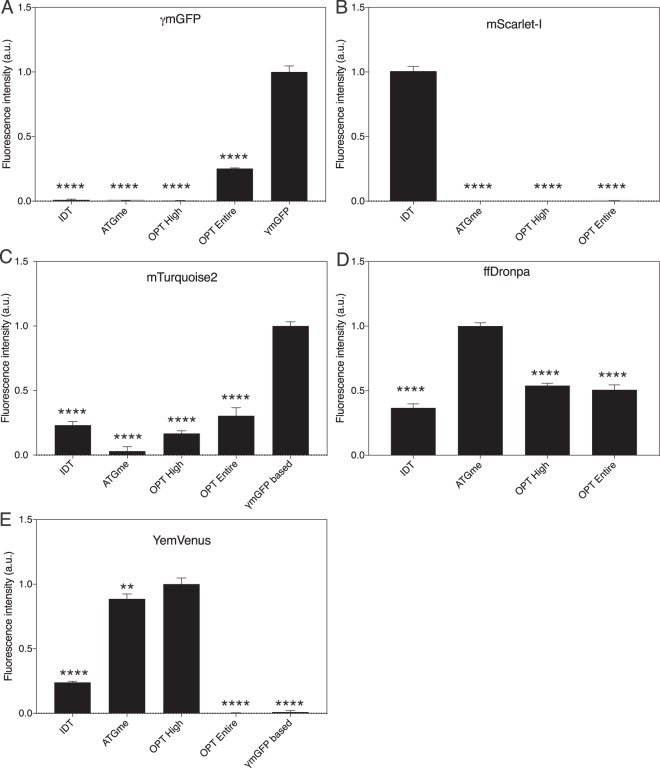
Fluorescence intensity measurements using a BD influx flow cytometer on *C. albicans* strains expressing different codon optimized versions of fluorescent proteins. Cells were grown overnight on low fluorescent medium, before bringing to an optical density at 600 nm (OD_600_) of 0.2 in fresh Low Fluorescent medium and grown to the exponential phase (4–5 hrs) before measurement. Data shown is corrected for wild type autofluorescence and normalized to the brightest codon variant. A) γmGFP B) mScarlet-I C) mTurquoise2 D) ffDronpa E) YemVenus. Asterisks denote significance level with **** corresponding to p < 0,0001 and ** corresponding to p < 0,01 when comparing with the brightest codon variant.

For each codon variant, 3 biological repeats were created and brightness was measured on a single cell level using FACS. The average values and SEM of each FP variant are plotted in Fig. 1, allowing for comparison of codon optimization strategies. Remarkably, these data show that there is no single optimization strategy that works optimally for all FPs. The existing codon sequence of γmGFP outperforms the sequences obtained through the other techniques by a wide margin (Fig. 1A). mTurquoise2, which is highly similar to γmGFP (96,2% sequence similarity), also shows the highest fluorescence for the coding sequence derived from γmGFP. Interestingly, although YemVenus is closely related to γmGFP (97,1% sequence similarity), it does not benefit from using the same codon sequence, but achieves maximum brightness following OPTIMIZER optimization with the self-constructed codon usage table of 5 highly expressed genes. Other methods also provide some expression, but significantly lower (P≤0,0042). The two non-*Aequorea* FPs, ffDronpa and mScarlet-I, show that yet other optimization strategies yield an optimal expression. ffDronpa expression benefits significantly (P < 0,0001) from the ATGme bulk optimization strategy, whilst still showing approximately half of the brightness using OPTIMIZER strategies. Within the mScarlet-I codon variants, there is almost no variation between optimizations, with only the IDT optimization showing a barely measurable fluorescence signal.

### Brightness assessment: fluorescence microscopy

**Figure 2:**
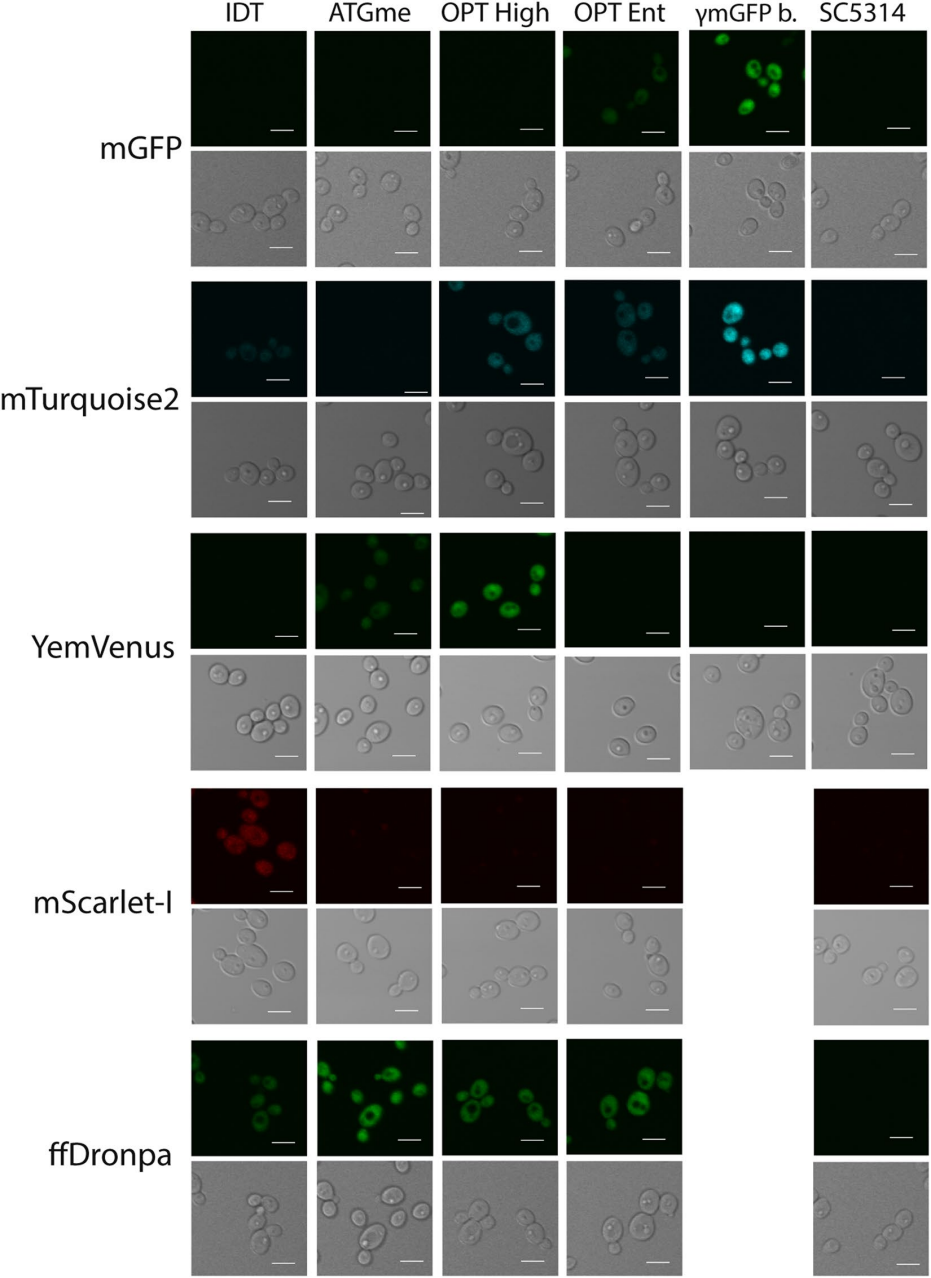
Images were taken with a Fluoview 1000 confocal microscope, using the appropriate excitation and emission filters for each fluorescent protein. Cells were grown overnight on low fluorescent medium, before bringing to an optical density at 600 nm (OD_600_) of 0.2 in fresh Low Fluorescent medium and grown to the exponential phase (4–5 hrs) before measurement. For each codon variant one representative strain is represented. Laser intensity is kept constant between codon variants of the same fluorescent protein and also between γmGFP and ffDronpa.

We next imaged cells expressing the different coding variants of each fluorescent protein using a confocal microscope, to assess their brightness under experimental conditions relevant to their intended use. These confocal images are shown in Fig. 2, and show the same fluorescence brightness distributions as the FACS data. We are therefore able to visualize even the dimmest coding variants of each fluorescent protein with a confocal microscope. Several optimization methods can thus yield visually perceivable FPs that can already be applied in experiments with highly (over-) expressing constructs. Nevertheless, our results clearly indicate that strong enhancements in expression can be achieved using the appropriate optimization strategy. As expected, the fluorescent strains show a clear localization to the cytosol.

### Application of codon-optimized fluorescent proteins

**Figure 3:**
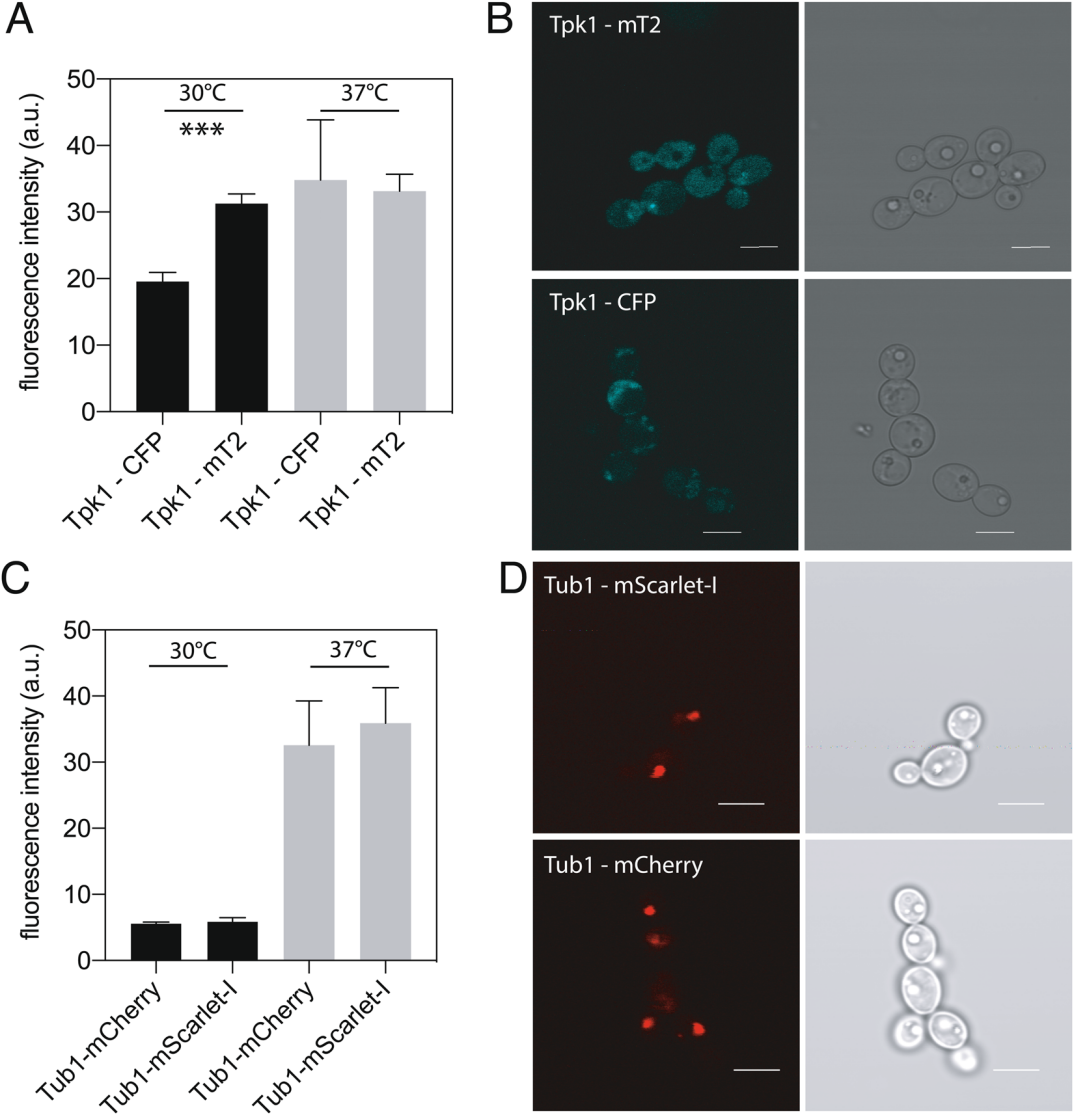
Fluorescence intensity measurements using a BD influx flow cytometer on *C. albicans* strains endogenously expressing (**A**) CFP or mTurquoise2 attached to Tpk1 and (**C**) mCherry or mScarlet-I attached to Tub1. Cells were grown overnight on low fluorescent medium and brought to an optical density at 600 nm (OD_600_) of 0.2 in fresh Low Fluorescent medium. Strains were allowed to grow to the exponential phase (4–5 hrs) at 30 °C or 37 °C, before measurement. Data shown is corrected for wild type autofluorescence. Asterisks denote significance level with ***corresponding to p < 0,001. B and D are confocal images taken with a Fluoview 1000 confocal microscope, using the appropriate excitation and emission filters for each fluorescent protein. Shown are representative images of Tpk1 – CFP and Tpk1 – mTurquoise2 (mT2) in panel B and representative images of Tub1 – mCherry and Tub1 – mScarlet-I in panel D.

The main application of these novel codon-optimized fluorescent proteins is intracellular imaging, preferably of targets using endogenous levels of expression. To assess if these novel proteins are an improvement or match the applicability of previously published FPs, we compared the performance of mTurquoise2 and mScarlet-I to respectively, CFP and mCherry. In Fig. 3A we directly compare the CFP against mTurquoise2 in a biologically relevant setting. For the cyan FPs attached to Tpk1 there is a significant (P = 0,0005) improvement of in cell fluorescence of mTurquoise2 compared to CFP at 30 °C. This difference between mTurquoise2 and CFP is absent at 37 °C. Results from fluorescence brightness assessment of the Tub1 FP-tagging with mCherry and mScarlet-I are depicted in Fig. 3C. mScarlet-I performs equally well as mCherry at both 30 °C and 37 °C. There is no clear improvement.

We also imaged the strains containing the endogenous tags using confocal microscopy. The results of these are depicted in Fig. 3. As was previously shown, the CFP and mTurquoise2 attached to Tpk1 (3B) show a localization to the cytosol^24^. Tubulin (Tub1) was tagged with mCherry and mScarlet-I and shows a punctate localization (3D) resembling the pattern observed with Tub1 – YFP reported by Gerami-Nejad and colleagues^25^.

### *In silico* analysis of RNA/DNA parameters

Understanding the fluorescence intensity differences between the optimization methods requires the availability of quantitative descriptors of the coding sequences. We calculated parameters that were previously shown to be important factors in the heterologous expression of genes. The first of these is secondary structure of the RNA, which is assessed as the Gibbs free energy released upon folding. It was shown that the presence of secondary structure in mRNA near the ribosome binding site negatively affects translation initiation and therefore protein levels^26^. More specifically, studies have shown that the folding energy of the full-length mRNA is not correlated with protein levels, whilst the folding energy of the first third of the mRNA sequence has a significant negative correlation with protein levels^26^.

The Codon Adaptation Index is a measure for the number of optimal codons used in the mRNA sequence, with 1 being the theoretical maximum. In *Escherichia coli*, it was shown that the CAI did not correlate with the expression level, but did correlate with faster growth, leading to the conclusion that overexpressing an mRNA sequence with low codon-optimality induces stress and hampers cell growth^26^.

It was shown that native mRNA sequences decode linking segments between structural domains with low-frequency codons. These rare codons stall the ribosome after a protein structural domain, which is then allowed to fold. This leads to a lower overall level of misfolded protein and less aggregation and degradation^27,28^. We calculated the amount of stretches containing 3 and more low-frequency codons, to assess the total amount of possible ribosome stalling. All calculated parameters are available in supplementary table 4.

**Figure 4:**
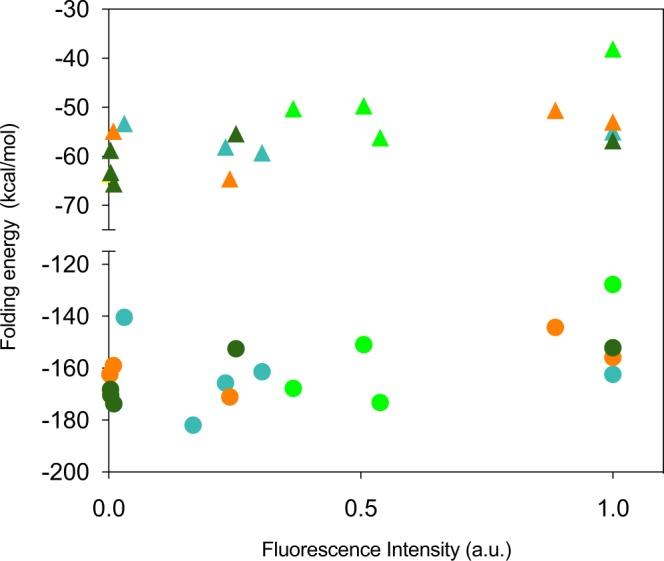
Scatter plot of the fluorescence intensity as measured using FACS versus (**A**) the folding energy of the structure from the full length (▲) and first 250nt (●) of the mRNA sequence as calculated using RNAfold. Colors denote different fluorescent proteins with orange for YemVenus, blue for mTurquoise2, Light green for ffDronpa and dark green for γmGFP.

We are unable to test for correlations between the quantitative parameters and the measured fluorescence intensity, because only 4 or 5 data points are present for each color variant. Visual inspection of the brightness-folding parameter plots did not reveal any clear trend. The most interesting data arose in the comparison of the RNA folding and the FACS fluorescence intensity data (Fig. 4), which may suggest a weak correlation between these parameters, potentially indicating that a lower amount of RNA folding and thus higher folding energy is beneficial for expression levels in *C. albicans*. This trend is visible in both the RNA folding of the full-length protein and the first third or 250 nucleotides of the fluorescent protein. However, this effect is not well established and further investigation would be required to clarify this further. None of the other calculated parameters display a clear relationship to the FACS fluorescence intensity.

## Discussion

Codon-optimization of genes encoding fluorescent proteins resulted in a significantly enhanced palette of fluorescent proteins. The difference in fluorescence brightness between optimizations ranged from 2 to 10-fold improvements, showing that codon usage is a large factor influencing expression levels in *C. albicans*. These improved fluorescence proteins all have their specific use in research. The original *Candida* specific γmGFP, which outperformed other GFP codon-variants created in this study, is already widely applied in localization studies. On the other hand, YemVenus and mTurquoise2 would also be interesting fusion proteins and may be very useful as an excellent fluorescence resonance energy transfer (FRET) pair in biosensors and protein-protein interaction studies. mScarlet-I is a useful protein in multi-color imaging, where spectral overlap between GFP, YFP and CFP can hamper the use of multiple colors. The last optimized fluorescent protein in this study is ffDronpa. This is a photoswitchable green fluorescent protein and is an essential tool in super-resolution microscopy techniques, thereby opening up the possibility of performing sub diffraction microscopy on cellular components of *C. albicans*. It is remarkable that there is no single optimization strategy that performs well on all fluorescent proteins.

We compared two previously published FPs to the novel codon-optimized alternative presented in this work. For mTurquoise2 there is a clear improvement in brightness compared to CFP when attached to Tpk1 at 30 °C. At 37 °C there is no difference in brightness, suggesting that the gain in brightness is diminished when there is increased expression of Tpk1 or that protein folding is impaired at higher temperatures for both mTurquoise2 and CFP^29^. In the case of mCherry and mScarlet-I, there is no significant difference in brightness when endogenously attached to Tub1. However, the rationally engineered mScarlet-I is functional in *C. albicans*, and has been shown to provide additional benefits in mammalian cell lines, such as a long excited state fluorescence lifetime and efficient use as a FRET acceptor^16^.

*In silico* analysis of the optimized sequences and calculation of the five previously described parameters suggests a trend between fluorescence intensity and folding energy of the full or first third of the protein (Fig. 4). Thereby indicating that both translation initiation and translation elongation can be hampered by RNA structures in the first part or the full protein. Whilst in *E. coli* only translation initiation was shown to be hampered by strong RNA folding.

Our results clearly show that there is currently is no generally-applicable technique for the optimization of heterologous expression. In fact, using just five different FPs and five optimization techniques, we find that each FP requires a different optimization technique to achieve optimal expression. In addition, we did not identify a predictive RNA parameter that could be used to assess overall expression levels of a novel FP. In the absence of such a generally-applicable and well-performing strategy for the optimization of *C. albicans* expression, we suggest performing several *in silico* optimizations using the online methods described above and experimental evaluation of the resulting expression levels. The RNA folding energies may hold some value when performing a relative comparison, though the supporting trend is weak at best.

Further research is necessary in this unexplored field of *C. albicans* heterologous gene expression, ideally resulting in development of a robust and generally-applicable optimization strategy. The cause of trends found in this study can be elaborated further and lead to insights in the development and translation of genomic tools from mammalian systems to *C. albicans*. Nevertheless, we present several optimized fluorescent proteins which have characteristics that will benefit imaging of *C. albicans*, such as ffDronpa for super-resolution microscopy. On the other hand, using endogenous tags, we assessed the overall cell brightness of mTurquoise2 and mScarlet-I compared to the already available options and saw a significant improvement for mTurquoise2 and a comparable performance of mScarlet-I compared to mCherry.

## Material and Methods

### In silico design

Sequences of the novel FPs mTurquoise2, mScarlet-I and ffDronpa were obtained from respectively, PDB with accession codes, 3ZTF, 5LK4 and addgene plasmid #101193. The DNA sequences encoding all fluorescent proteins used, were designed *in silico* using four different web-based codon optimization tools. The OPTIMIZER tool (http://genomes.urv.es/OPTIMIZER/) requires the amino acid sequence of the FP as an input and was applied with two different codon usage tables^21^. One table was obtained from the Codon Usage Database (http://www.kazusa.or.jp/codon/) and was tabulated using the entire genome of *C. albicans*^30^. The other codon usage table was determined by five highly expressed genes, *RPL29, RPL32, RPL39, ACT1* and *ENO1*, and was tabulated using the CountCodon program (http://www.kazusa.or.jp/codon/countcodon.html). The IDT codon optimization tool (https://eu.idtdna.com/CodonOpt) within the SciTools^®^ web application requires the amino acid sequence of the FP as an input and the selection of *C. albicans* in the dropdown menu to determine the codon usage table. ATGme (http://atgme.org/) requires the input of a DNA sequence, the DNA sequence obtained from the IDT tool was inserted here. The codon usage table was obtained from the Codon Usage Database^23^. The Bulk Optimize feature was selected, after which we manually replaced all rare codons with the most optimal codon, acquiring a sequence with the highest Codon Adaptation Index (CAI). Another optimization strategy was based on the codon usage and sequence of γmGFP. We manually optimized YemVenus and mTurquoise2 by taking the DNA sequence of γmGFP and inserting the specific mutations to acquire YemVenus and mTurquoise2. This method was not applicable to mScarlet-I and ffDronpa because they are dissimilar to the GFP protein family due to the fact that they are not derived from *Aequorea Victoria*.

### Generation of FP overexpression strains

The obtained DNA sequences were synthesized as gBlocks (IDT) or BioXp^TM^ Tiles (Synthetic Genomics Inc.) with flanking sequences allowing Gibson cloning. YemVenus and γmGFP genes were PCR amplified from the previously published pFA6 plasmids^13^ and listed in supplementary table 1. The CIp10 plasmid, where the *URA3* marker was previously replaced by a *NAT1* gene conferring resistance to nourseothricin, was digested with high fidelity PstI and NheI (NEB) before insertion of the synthetic DNA construct or PCR product using NEBuilder® HiFI DNA assembly Cloning kit. After transformation of the StuI-linearized CIp10 plasmid into SC5314, we performed selection with nourseothricin.

### Selection of strains with single insertion of CIp10 using quantitative PCR

DNA was extracted using phenol/chloroform/isoamyl alcohol (PCI) followed by ethanol precipitation. DNA was diluted to 0.5 ng/μL and checked for a single genomic DNA integration of the plasmid using quantitative PCR. 2.5 ng/μL of DNA was utilized in the standard protocol of Promega with GoTaq® qPCR Master Mix, CXR dye and primers listed in supplementary table 2 at a final concentration of 1 μM on the StepOnePlus Real-Time PCR system from Thermo Fisher Scientific. QPCR analysis was performed with Biogazelle Qbase+ software. Single copy number data is available in supplementary table 3.

### Generation of endogenous tags

For the endogenous tagging of Tpk1 we constructed a pFA6-based plasmid containing mTurquoise2. From the mTurquoise2 gBlock we amplified a Gibson insert using primers listed in supplementary table 1. The pFA6 plasmid containing a histidine autotrophy marker was digested with PstI and SmaI before insertion of mTurquoise2. A pFA6-based plasmid containing CFP was already available^25^. Using PCR with primers (supplementary table 1) on these pFA6 plasmids, we created linear fragments with 100 bp overhang for recombination with the end of the *TPK1* gene, without the stop codon, and the start of its terminator. Between the *TPK1* gene and the start of the FP, we included a linker sequence containing a triple repetition of glycine and alanine. For the endogenous tagging of Tub1 we utilized a fusion PCR approach using primers listed in supplementary table 1. We created two separate PCR fragments one containing the FP, in this case mScarlet-I or mCherry from the gBlock, and the second containing the nourseothricin selection marker from the pFA6 plasmid. These fragments were then fused containing 100 bp of overhang for recombination with *TUB1* and the same linker as reported above, and transformed into *C. albicans*.

### Strains and growth conditions

All plasmids were transformed into the lab strain SC5314^31^. The strains were grown overnight on Low Fluorescent medium containing 0,69%(w/v) Yeast Nitrogen Base without amino acids, folic acid and riboflavin, 0,25%(w/v) Ammonium sulphate and 2%(w/v) glucose before bringing to an optical density at 600 nm (OD_600_) of 0.2 in fresh Low Fluorescent medium and grown to the exponential phase (4–5 hrs) before measurement of fluorescence with Fluorescence Activated Cell Sorting (FACS) or microscopy.

### FACS analysis of fluorescence intensity

Single cell fluorescence measurements were performed using the BD Influx^TM^ Cell Sorter at 100 000 cells per strain, with 3 biological repeats for each codon variant. Green and yellow FPs were excited using 488 nm from a laser source and emission was recorded with a 550LP combined with a 530/40 filter. mScarlet-I and mCherry were excited using a 561 nm laser and emission was recorded through a 685LP combined with a 670/30 filter. mTurquoise2 and CFP were excited using a 405 nm laser and emission was recorded through a 455LP and a 470/20 filter. Obtained FACS data were analysed using FLOWJO^®^ to acquire mean fluorescence intensities for each strain. Fluorescence intensity was then corrected for the autofluorescence of the wild type strain SC5314 in the respective channels. Mean fluorescence was then normalized to the brightest codon variant of each fluorescent protein.

### Confocal microscopy

Cells were grown in an identical manner as for FACS measurement. Cells were imaged using a Fluoview FV1000 confocal laser scanning microscope. mTurquoise2 and CFP were excited with 458 nm light from an argon laser and emission was recorded through a bandpass filter BA480–495.

γmGFP and ffDronpa were excited with the same intensity of 488 nm laser and emission was detected through a bandpass filter BA505–565.

YemVenus was excited with 515 nm laser light and emission was detected through a bandpass filter BA535–565. mScarlet-I and mCherry were excited using a 559 nm laser and emission was recorded through a bandpass filter BA575–675.

### In silico analysis of DNA/RNA parameters

To assess the relation between the sequences based on the different optimization strategies and the resulting gene expression, as monitored by fluorescence intensity, we calculated several parameters. The Codon Adaptation Index (CAI) was analysed using the OPTIMIZER program with the codon usage table of the entire genome as a reference set. The GC content was provided by the ATGme tool. Using the same ATGme tool we were able to count the number of consecutive codons (2 or more) with a genome wide frequency lower than 15%. The Minimum Free Energy (MFE) of the folding of the RNA sequence was calculated for the entire protein coding region and the first 250 nucleotides of this region using the RNAfold program^32^. Codon frequencies were calculated using the codon plot tool within the sequence manipulation suite^33^.

### Statistical analysis

Differences in fluorescence intensity between codon variants were assessed using a one-way ANOVA with a two-sided Tukey’s multiple comparison in Prism 7. Significant differences between the brightest variant and all of the other variants are depicted in Fig. 1. Differences in fluorescence intensity between CFP and mTurquoise2 or mScarlet-I and mCherry were assessed with a Student’s t-test.

*Data availability*. FACS data is available at FlowRepository under ID: FR-FCM-Z2W5. Synthetic nucleotide sequences and accession numbers are available in supplementary table 4.

## Supplementary information

Supplementary Tables 1–4.
